# Gene expression within the periaqueductal gray is linked to vocal behavior and early-onset parkinsonism in Pink1 knockout rats

**DOI:** 10.1186/s12864-020-07037-4

**Published:** 2020-09-17

**Authors:** Cynthia A. Kelm-Nelson, Stephen Gammie

**Affiliations:** 1 Department of Surgery, Division of Otolaryngology-Head and Neck Surgery, University of Wisconsin-Madison, 1300 University Avenue, 483 Medical Sciences Center, Madison, WI 53706 USA; 2 Department of Integrative Biology, University of Wisconsin-Madison, Madison, WI USA

**Keywords:** Parkinson’s disease, Rat, Pink1, RNA sequencing, WGCNA, Bioinformatics, Vocalization, Periaqueductal gray

## Abstract

**Background:**

Parkinson’s disease (PD) is a degenerative disease with early-stage pathology hypothesized to manifest in brainstem regions. Vocal deficits, including soft, monotone speech, result in significant clinical and quality of life issues and are present in 90% of PD patients; yet the underlying pathology mediating these significant voice deficits is unknown. The *Pink1*−/− rat is a valid model of early-onset PD that presents with analogous vocal communication deficits. Previous work shows abnormal α-synuclein protein aggregation in the periaqueductal gray (PAG), a brain region critical and necessary to the modulation of mammalian vocal behavior. In this study, we used high-throughput RNA sequencing to examine gene expression within the PAG of both male and female *Pink1*−/− rats as compared to age-matched wildtype controls. We used a bioinformatic approach to (1) test the hypothesis that loss of *Pink1* in the PAG will influence the differential expression of genes that interact with *Pink1*, (2) highlight other key genes that relate to this type of Mendelian PD, and (3) catalog molecular targets that may be important for the production of rat vocalizations.

**Results:**

Knockout of the *Pink1* gene resulted in differentially expressed genes for both male and female rats that also mapped to human PD datasets. Pathway analysis highlighted several significant metabolic pathways. Weighted gene co-expression network analysis (WGCNA) was used to identify gene nodes and their interactions in (A) males, (B) females, and (C) combined-sexes datasets. For each analysis, within the module containing the *Pink1* gene, *Pink1* itself was the central node with the highest number of interactions with other genes including solute carriers, glutamate metabotropic receptors, and genes associated with protein localization. Strong connections between *Pink1* and *Krt2* and *Hfe* were found in both males and female datasets. In females a number of modules were significantly correlated with vocalization traits.

**Conclusions:**

Overall, this work supports the premise that gene expression changes in the PAG may contribute to the vocal deficits observed in this PD rat model. Additionally, this dataset identifies genes that represent new therapeutic targets for PD voice disorders.

## Background

Parkinson’s disease (PD) is the second most common degenerative disorder affecting nearly 10 million people worldwide [1]. The hallmark pathology of PD is death of dopaminergic neurons in the substantia nigra; however, pathology outside of dopamine loss often precedes clinical presentation of limb motor symptoms [2–4]. Vocal communication deficits, including hypokinetic dysarthria [5–8], are common and during the course of disease progression, over 90% of individuals present with these deficits. These signs negatively influence overall health, social interactions, employment, and quality of life [6, 7, 9, 10]. Despite this considerable clinical issue, the underlying central nervous system pathology that contributes to vocalization deficits in PD is poorly understood and understudied.

The standard pharmacological and surgical treatments aimed at restoring dopamine loss are generally ineffective for voice dysfunction which further suggests that vocal deficits are linked to other, unknown CNS pathologies [11–13]. Research suggests that PD in the central nervous system progresses in a caudal-to-rostral direction, with the brainstem impacted earlier than cortical regions [3, 4]. The periaqueductal gray (PAG) is a phylogenetically conserved brainstem nucleus implicated in the modulation and production of mammalian vocalizations [14–16]. Specifically, descending projections from the PAG modulate the motoneurons in the brainstem that control vocal fold adduction and respiration coordination [17, 18]. Additionally, the PAG is interconnected with the social behavior network [16] and the ascending cortical and limbic projections from the PAG are hypothesized to influence the motivation or emotional intent of species-specific vocalizations [19–21]. In humans, functional imaging studies demonstrate that the brainstem PAG is also involved in the circuitry and control of speech [22], and appears to be involved in the speech patterns of individuals with PD (where PAG connectivity is correlated to speech loudness) [23].

Genetic rodent models of PD are useful because they mimic aspects of idiopathic PD and translate with an early and progressive disease manifestation. The *Pink1*−/− rat, related to the PARK6 phenotype of human familial PD, demonstrates early motor deficits including changes to ultrasonic vocal production [24, 25]. Specifically, data show that male and female *Pink1*−/− rats exhibit early (2 months of age) ultrasonic vocalization changes to intensity (loudness) compared to non-affected wildtype (WT) control rats [24, 26]. Additional acoustic deficits include changes to frequency range in male rats at 8 months of age [24]. Moreover, WT female conspecifics show decreased motivation to approach male *Pink1*−/− vocalization stimuli, suggesting that these deficits impair the social communication function [27]. More recent data suggest that these vocal deficits are not rescued by pharmacological dopamine replacement (levodopa) [28], but can be, as in humans, modulated by a vocal-exercise therapy [29]. In general, these deficits recapitulate findings observed in humans with PD, irregular vocal qualities that impact the ability to effectively communicate. In rats, the stimulation of the PAG increases production of vocalizations [15]. Further, lesions to the PAG result in muteness [17], and its neurotransmitters and projections are connected to the peripheral structures (vocal fold) of vocalization [30]. At 8 months, *Pink1*−/− male rats exhibit significant aggregation of insoluble α-synuclein, a pathological marker of PD [31]. Moreover, PAG protein aggregation has also been implicated in vocal dysfunction in a mouse α-synuclein over expression model [32]. Together, these data contribute to the working hypothesis that pathological changes in the PAG may account for aspects vocal dysfunction observed in the *Pink1*−/− PD rat.

Large scale gene expression analysis in post-mortem PD tissue and normal controls has led to the generation of detailed databases of the gene expression underlying the disease (see metanalysis [33]) and has contributed to the knowledge of genetic variants underlying the disease [33]. Many of these studies are focused on the midbrain substantia nigra [34, 35], frontal lobe [36], and more recently blood-brain [37] and gut biomarkers [38]. A primary advantage of a genetic model is the homogeneity of the sample that cannot be assessed with human post-mortem tissue. Because the etiology of PD is diverse and the pathology of vocal deficits is significantly understudied, in this study we used RNA sequencing to characterize differentially expressed genes within the PAG in the *Pink1*−/− rat compared to age-matched wildtype controls at 8 months of age. We tested the specific hypotheses: [1] loss of *Pink1* in the PAG will influence expression of genes that interact with *Pink1* [2]; loss of *Pink1* will emphasize other genes that relate to PD; and [3] behavioral and bioinformatic approaches to data analysis will identify molecular targets important for rat vocalization. To further validate the *Pink1* rat model, we compared our dataset to existing human databases to identify possible therapeutic targets for PD voice deficits.

## Results

### Differential gene expression and KEGG pathway analysis between genotypes and sex

We first evaluated differential gene expression in the PAG between *Pink1*−/− and WT rats; *Pink1* was the most significant downregulated gene in both males and females. Using the *p* value as a cutoff threshold (*p* < 0.05), there were 675 genes (520 annotated) within males and 1155 genes (973 annotated) within females found to be differentially expressed. Comparison of the top 500 downregulated genes showed a significant overlap between sexes (76 genes, hypergeometric *p* value< 0.0001). The gene overlap between the 500 upregulated gene list was also statistically significant (63 genes, hypergeometric *p* value < 0.0001).

**Fig. 1:**
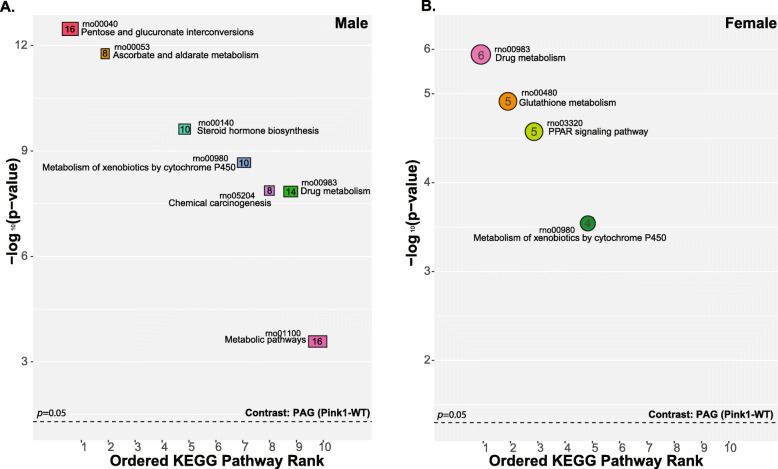
Ordered KEGG pathway rank in males and females. Significant metabolic pathways in (A) male and (B) female periaqueductal gray RNA-sequencing datasets (contrast *Pink1*−/−: WT). RNO = KEGG pathway entry for *Rattus norvegicus*. Significantly enriched KEGG pathways are ordered from most to least significant. The number of genes in the specified KEGG pathway are denoted by the size of a square (males) or circle (females) and the specific number of expressed genes for each sex, respectively. The color filling each circle corresponds to the specific KEGG pathway description

Sex-specific enrichment analysis was used to compare the gene sets to existing KEGG network datasets (Fig. 1). Briefly, in males, pathways (*genes*) of interest include pentose and glucuronate interconversions (*Akr1b10, Ugt1a9*), and glycine, serine and threonine metabolism (*Psat1, MaoB*) (Fig. 1A). In females, glutathione metabolism (*Gstm3, Sms*), PPAR signaling (*Ubc, Slc27a6*), and metabolism (*Tpmt, Gstm3*) pathways were identified (Fig. 1B). Overlapping pathways include drug metabolism and metabolism of xenobiotics by cytochrome P450. There were more highlighted KEGG pathways in males [7] compared to females [4].

**Fig. 2:**
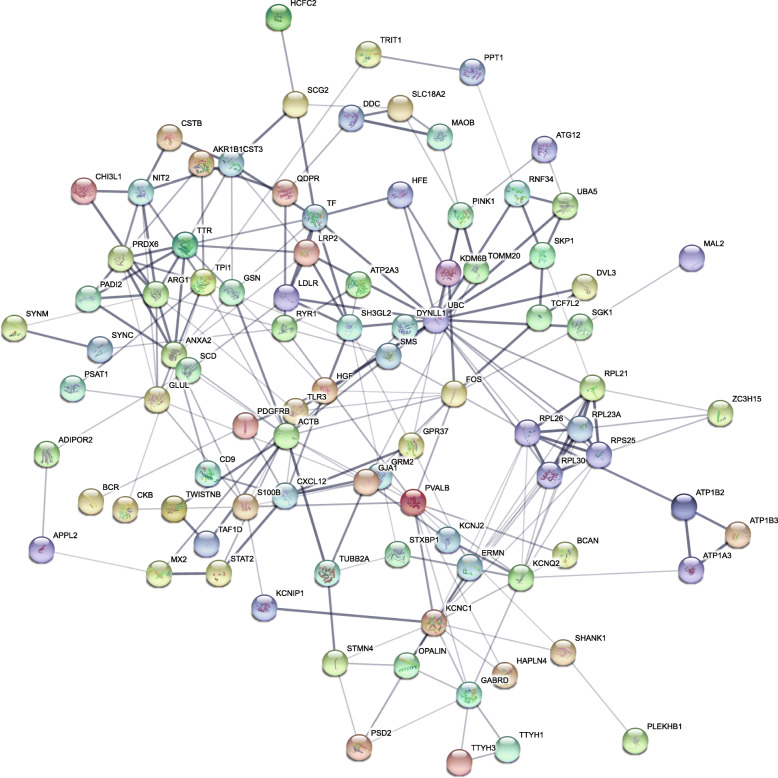
Protein-protein interaction networks. STRING model of protein interactions via genes that are commonly up and down regulated in male and female *Pink1*−/− rats and in humans with PD. Nodes are proteins and edges indicate protein interactions

Enrichment analysis (using Enrichr) of the downregulated genes in females identified significant matches to genes downregulated in the CNS with PD in humans as well as PARK2 and PARK7 knockdown in human neuronal or tumor cell lines, suggesting the *Pink1*−/− rat model recapitulates genetic and idiopathic PD in humans (Table 1). Likewise, upregulated genes in *Pink1*−/−females match upregulated genes in the CNS in humans with PD. Similar matching of the *Pink1*−/− male brain gene expression with that found in humans with PD was identified. A weighted graph was created using the identified protein-protein interactions (Fig. 2) obtained from the STRING database. Initially, a human-rat list of 160 genes was entered into STRING and the default cutoff of gene-gene connectivity of 0.4 was used to exclude gene with little or no connectivity. The remaining genes were ranked based on the number of interactions with other unique genes and the top 83 genes with high interactions are plotted within the figure.

**Table 1:** Example of directional overlap with human PD datasets. Grey text is associated with non-significant *p* values

Current Rat Data Set	Human Data Sets	Adjusted ***p*** value	Overlapping Gene List
Female, downregulated	Parkinson’s disease CNS: downregulated GSE19587 [34] Region: inferior olivary nucleus Sex: Males and females Subjects: PD vs Control	0.00030	*Rpl30; Rpl21; Plekhb1; Gdf1; Hsd17b4; Adipor2; Tf; Tspan8; Kif5c; Sgk1; Rps27a; Cryab; Rps24*
	PARK7 knockdown Peripheral: downregulated GSE5519 [39] DJ-1 cell lines: H157 non-small-cell lung carcinoma cells	0.020	*Hsp90aa1; Rpl21; Psmd14; Pih1d1; Pomp; Hspe1; Cox5a; Tomm20; Cox6b1; Hspd1; Mob4; Dnaja1; Efr3a; Psma4; Ugp2; Tomm7; Uqcrfs1; Rps24; BIrc2; Chmp5*
Female, upregulated	Parkinson’s disease CNS: upregulated GSE7621 [40] Region: Substantia nigra Sex: Males and Females Subjects: PD vs normal control	0.00039	*Tiam1; Tcf7l2; Gja1; Ddit4; Clstn1; Irs2; Actb; Scn1b*
	PARK2 knockdown Tumor cells: upregulated GSE50864 [41] Glioma cell lines: SNB19 and SF539	0.00012	*Gja1; Ednrb; Atxn1; Temn4; Col5a1; Ctnnd2; Lrp4; Cd59; Megf8; Mylk*
*Male*, downregulated	Parkinson’s disease CNS: downregulated GSE19587 [42] Region: inferior olivary nucleus Sex: Males and females Subjects: PD vs Control	0.061	*Eef1a1; Slc14a1; Tuba1c; Tf; Sparc; Rps3a; Sgk1; S100b; B2m; Ppia; Gfap*
	PARK7 knockdown CNS: downregulated GSE17204 [43] DJ-1 silenced neuroblastoma vs control cells	0.019	*Gpr37; Sema3c; Stxbp1; F2r; Lrp4; Atp1a3; Esrrg; Abcc10; Lamp5; Qdpr; Trim9; C7; Dpysl3; Anxa6; Spock1; Spry1; Pfkm*
*Male*, upregulated	Parkinson’s disease CNS: Upregulated GSE7621 [34] Region: inferior olivary nucleus Sex: Males and females Subjects: PD vs Control	1.0	*Qdpr; Cdkn1b; Slc24a3; Usp54; Atp1a3; Spock1; Stom; Rpl23a; Sh3gl2; Pvalb*
	Park7 knockdown CNS: downregulated GSE17204 [43] DJ-1 silenced neuroblastoma vs control cells	0.12	*Dhfr; Nfasc; Pgbd5; Epas1; Atp1a3; Stmn4; Slc27a2*

Downregulated genes of interest in females include genes involved in RNA binding and transcription (*Hsp90aa1; Pih1d1; Hspe1*), metabolism/mitochondria functions (*Hsd17b; Adipor2; Cox5a; Cox6b1; Ugp2, Uqcrfs1*), iron (*Tf; Hspe1*), vesicle signaling (*Tspan8; Kif5c*), ubiquitin (*Rps27a; Psmd14; Hspd1; Dnaja1; BIrc2*), ribosomal proteins (*Rpl30, Rpl21, Rps24*), and protein activity (*Tomm20, Tomm7, Efr3a)*. In males, downregulated genes of interest that matched human Parkinson disease sequencing datasets included ubiquitin (*Gpr37, Trim9)*, axon guidance (*Sema3c)*, and syntaxin (*Stxbp1*).Notable upregulated genes that overlapped between the *Pink1*−/− female rat and human datasets included genes involved in neuron signaling (vesicle (*Cd59)* sodium channel activity/membrane depolarization (*Scn1b),* calcium signaling (*Ednrb; Mylk),* synapse organization *(Lrp4)).* There were associations with amyloid-beta binding (*Clstn1, Cryab, Atp1a3)* a neurological feature of protein aggregation in Alzheimer’s disease.

### Gene interactions with the Pink1 gene using WGCNA

A primary goal of this work was to determine which genes interacted with *Pink1*. The annotated gene lists were run with WGCNA and data is presented in three ways: [1] male (Supplementary Table 1), [2] female (Supplementary Table 2), and a [3] combined-sexes approach (Supplementary Table 3). A number of modules (male = 29 modules; female = 26, combined sexes = 9) were generated; these tables can be sorted by module and trait (Supplementary Tables 4 and 5).

The following WGCNA modules included the *Pink1* gene: for males the red module, females the lightcyan module, and for the combined-sexes the pink module. The sex-specific analysis of red and lightcyan modules that included *Pink1* showed interactions with a different subset of genes, suggesting a possible sex-specific difference. For all three modules, *Pink1, Krt2, Tert* were the only genes that overlapped. A list of genes that appeared in at least two of the modules can be found in Table 2.

**Table 2:** List of gene candidates appearing in modules with *Pink1* in more than one condition (male, female, combined-sexes)

Gene Abbreviation	Gene Name
*Pink1*	*PTEN induced kinase 1*
*Krt2*	keratin 2
*Hfe*	homeostatic iron regulator
*Srd5a1*	steroid 5 alpha-reductase 1
*Pih1d1*	PIH1 domain containing 1
*Mocos*	molybdenum cofactor sulfurase
*Tm4sf4*	transmembrane 4 L six family member 4
*Mylk3*	myosin light chain kinase 3
*Lrrc63*	leucine rich repeat containing 63
*Akr1b8*	aldo-keto reductase family 1, member B8
*Tert*	telomerase reverse transcriptase
*Cyyr1*	cysteine and tyrosine rich 1
*Hpd*	4-hydroxyphenylpyruvate dioxygenase
*Tlr3*	toll-like receptor 3
*Apol3*	apolipoprotein L, 3
*Siglec5*	sialic acid binding Ig-like lectin 5

**Fig. 3:**
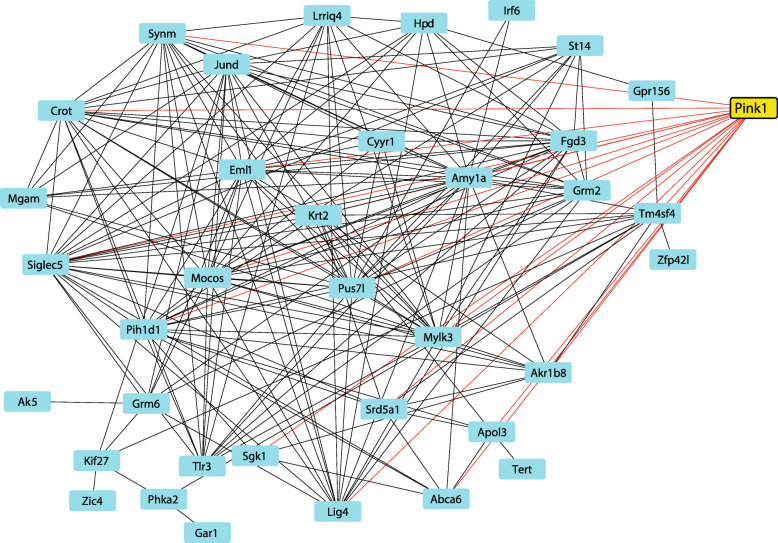
Characterization of the combined-sexes MEPink WGCNA module. The interaction network of the differentially expressed genes was visualized using Cytoscape 3.3.0 software. Data were plotted using weight (level of significance) as a factor. Only annotated genes are plotted from the combined sexes dataset (37 nodes, 203 edges). *Pink1* (yellow) was the top gene with the highest number of significant connections. Lines represent significant correlations between two genes. Red lines represent significant correlations between *Pink1* and other module genes; black lines are connections between module genes (note: node colors do not correlate to the WGCNA module color)

To determine the genes and their functions that interact with *Pink1*, the 45 genes that were in combined sexes pink module (displayed in Fig. 3) were put into the gene enrichment analysis tool to evaluate this gene list against preexisting data sets. Using Enrichr to evaluate gene ontology and biological processes, several areas of enrichment were identified including establishment of protein localization (*Tert, Pih1d1*) and glutamate receptor signaling (*Grm2, Grm6).*

### Female ultrasonic vocalization behavior and WGCNA

**Fig. 4:**
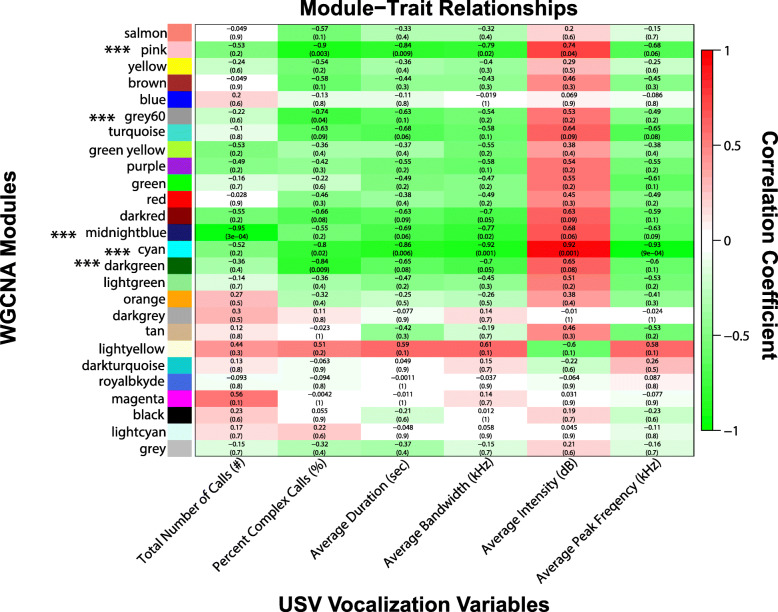
Module-trait relationships. Detected statistical associations between expression profiles for each of the WGCNA identified modules (y-axis) and female frequency modulated vocal behavior (x-axis). Boxes contain correlation coefficients (*p* values) where Red = strong positive correlation; Green = strong negative correlation). The genes comprising each module are listed in Supplementary Tables 3 and 4. *** indicate modules that correlated significantly with vocalization variables (see Table 3)

Previously recorded female vocalization data [26] from WT and *Pink1*−/− rats was used in a WGCNA to identify vocalization-associated modules (Fig. 4). Using the 26 modules for female rats, the statistical links with acoustic and non-acoustic variables were identified (Table 3). Vocal modules with the top significant trait relationships included cyan, pink, midnightblue, darkgreen and grey60.

**Table 3:** Significant female vocalization modules

USV parameter	Module	***p*** value
Average FM Duration (s)	cyan	0.0059
	pink	0.0094
	midnightblue	0.057
Average FM Bandwidth (kHz)	cyan	0.0013
	pink	0.019
	midnightblue	0.025
Average FM Intensity (dB)	cyan	0.001
	pink	0.037
Average FM Peak frequency (kHz)	cyan	0.00094
Number of calls (#)	midnightblue	0.00025
% Complex Calls	pink	0.0027
	darkgreen	0.0086
	cyan	0.018
	grey60	0.036

**Fig. 5:**
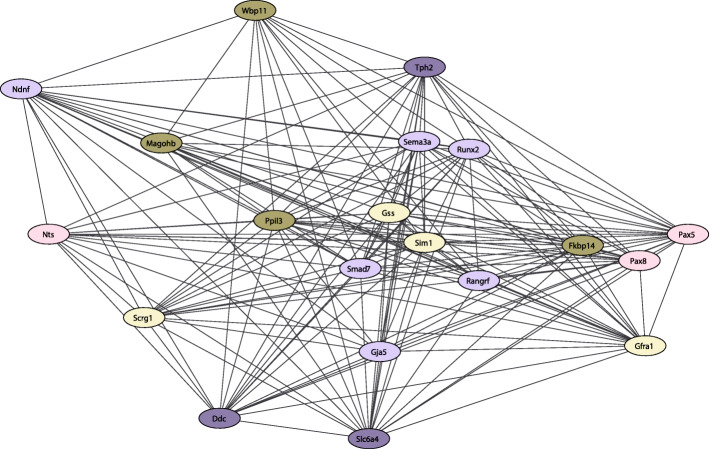
Gene-vocalization links. MeCyan gene module is the most positively correlated module with all female rat acoustic properties. ENRICHR gene enrichment software was used to examine gene pathways within this module, as presented here. Data were plotted using weight as a factor using Cytoscape 3.3.0 software. The most significant connections are plotted using weight as a factor; lines represent significant correlations between genes. Colors are indicative of the same enriched pathway: Dark purple (monoamine transmembrane transporter activity; dopamine transmembrane transporter activity; indolalkylamine metabolic process): *Slc6a4, Ddc, Tph2*; Light yellow (nervous system development, axon guidance/axonogenesis): *Gss, Gfra1, Sim1, Scg1*; Brown (RNA splicing; mRNA processing; peptidyl-prolyl cis-trans isomerase activity): *Ppil3, Fkbp14, Wbp11, Magohb*; Light Purple (neuron migration, axon guidance; generation of neurons and depolarization): *Sema3a, Ndnf, Runx2, Smad7, Gja5, Rangrf*; Light Pink (neuropeptide, cell signaling; transcription): *Pax8, Pax5, Nts*

**Fig. 6:**
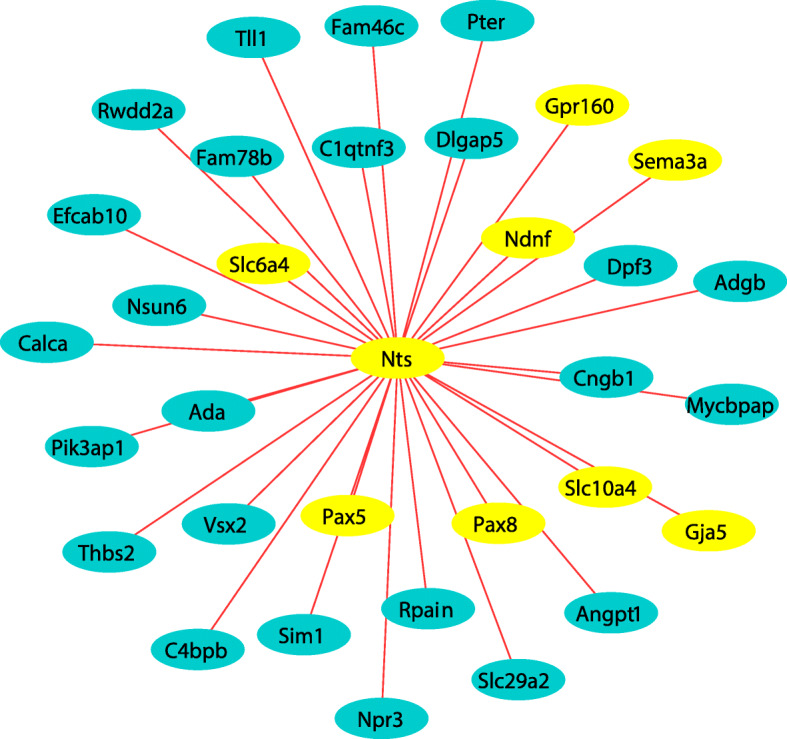
Neurotensin connections. The Nts node and its connections (from the MECyan module). Data were plotted using weight as a factor using Cytoscape 3.3.0 software. The most significant connections plotted using weight as a factor; red lines represent significant correlations between genes. Genes in yellow represent genes of interest, other connecting genes are in blue

Because the cyan module was significantly linked with all vocal acoustic parameters of the ultrasonic calls, including duration, bandwidth, intensity, and peak frequency, as well as the percent of complex calls we focused enrichment analysis on this specific module (all other modules are sortable within Supplementary Tables 3 and 4). Performing gene enrichment analysis on the gene list from the cyan module, the top biological pathways were the regulation of membrane depolarization (GO:0003254) and indolalkylamine metabolic process (GO:0006586). Other pathways of interest include monoamine transport (GO:0015844); generation of neurons (GO:0048699); dopamine transmembrane transporter activity (GO:0005329); nervous system development (GO:0007399), axon guidance (GO:0007411); mRNA splicing (GO:00003898); and neuropeptide hormone activity (GO:0005184). Figure 5 represents top pathways (genes) of interest within the cyan module. Further evaluation of the *Nts* node suggests several biologically relevant connections (Fig. 6) including *Slc6a4*, *Slc10a4*, *Ndnf, Pax5, Pax8, Sema3a, Gja5,* and *Gpr160.*

## Discussion

In general, the understanding of Mendelian inherited forms of PD is limited, but necessary in order to provide insight into the genetic nature of the disease. At 8 months of age, the *Pink1*−/− genetic rat model of PD exhibits observable limb and cranial sensorimotor dysfunction including deficits in ultrasonic vocal communication that co-occur with aggregated α-synuclein in the PAG. The aim of the present study was to identify differentially expressed genes using high throughput RNA-sequencing in the brainstems of *Pink1−/−* male and female rats as compared to their respective age-matched wildtype (WT) controls and use bioinformatic approaches to further validate this PD model and highlight specific gene targets that may modulate vocal behaviors in the rat. This study successfully generated datasets of genes including lists of differentially expressed genes in both males and females; there was statistically significant overlap between both sexes. The upregulated and downregulated genes showed similarity to human Parkinson disease studies as well as PARK gene models which provides further validity at the gene-level. WGCNA bioinformatic approaches highlighted several gene pathways that may be important for vocalization. Together these findings highlight new directions for targeting vocal biology pathways.

Phenotypically, motor signs of PD manifest differently in males compared to female *Pink1*−/− rats [26]. For example, females do not show the classical limb motor deficits in tapered balance beam or cylinder tests compared to progressive slowness and increased number of errors in males. Both females and males show decreases in vocal loudness; however, males also have changes to other vocal variables including reductions in peak frequency and bandwidth. In this study, the comparison of sequencing runs in males and females yielded strong significant relationships between sexes suggesting that the removal of the *Pink1* gene in both sexes yields similar genetic expression in the PAG. What contributes to the observed differences in behavioral phenotypes is unknown and should be addressed in future work. In normal cases, *Pink1* has a protective role against mitochondrial quality, dysfunction, and mitophagy by activating a mitochondrial damage-response signaling pathway. The Pink1: Parkin: Ubiquitin interaction has been well studied [44]. Here, we have recapitulated the important relationship between *Pink1* and *Ubc* in both male and female gene datasets as *Pink1* and *Ubc* were the top genes within the same-sex module pink. Activation of this pathway results in selective autophagy. Thus, loss of function mutations in this process ultimately lead to increases in oxidative buildup and mitochondrial damage which has been previously reported [42, 45–48].

Interestingly, comparison of the modules that include *Pink1* yielded different co-expressed genes; the only overlapping genes were *Pink1*, *Kert2*, and *Hfe*. The interaction between *Pink1* and *Hfe* suggests a mitochondrial: iron link, that has not previously been investigated in this particular in vivo model. *Hfe* expression modulates cellular iron absorption (reviewed in [49]), with a strong correlation to PD. For example, abnormal iron concentrations in the basal ganglia are hypothesized to induce PD symptoms [50]. Previous reports in the *Pink1*−/− rat suggest the presence of mitophagy including striatal mitochondrial proteomic alterations, and challenges within the mitochondria respiratory system. Iron accumulation within the neuron may be a mechanism of neurodegeneration and additional changes with age (i.e. disease progression). Pathway analysis from the female data showed significant genotype contrasts for the glutathione pathway [51] which is consistent with the strong links between iron accumulation, mitochondrial dysfunction (removal of impaired mitochondria), and resulting oxidative inflammation in the central and peripheral nervous system [52]. *Hfe* polymorphisms also affect cellular glutamate levels in cell lines [53]; glutamate receptor alterations observed in this study are discussed below. Our data suggest the *Pink1*−/− rat is a representative model of these types of complex interactions.

Activation of the PAG has significant effects on neurotransmitter release (reviewed in [54]), including GABAergic and glutamatergic signaling which suggests a role for multiple neurotransmitter systems in vocal biology [55–57]. Previously, we have shown that glutamate decarboxylase 1 (*Gad1*) in the PAG is significantly reduced in *Pink1*−/− rats [58] and our most recent work suggests that modulation of the GABA neurotransmitter represents therapeutic targets for rescuing vocalization. For example, within the VTA of *Pink1*−/− rats *Gad* gene expression is upregulated with an intensive behavioral vocalization intervention [59]. In this dataset, there are several glutamatergic pathways (genes) that were identified with gene enrichment analysis from the combined sex pink module. Two genes, *Grm2* (codes for L-glutamate) and *Grm6* (glutamate metabotropic receptor 6) are of particular interest as metabotropic glutamate receptors have been hypothesized to be promising anti-Parkinsonian drugs [60]. And, glutamate remains an important excitatory neurotransmitter for the production of vocalizations. For example, in the squirrel monkey, glutamate induces vocalizations when injected into the periaqueductal gray [57], and blocking glutamate receptors suppresses vocalization (as well as locomotion) in mice [61]. Thus, compounds that promote glutamate receptor expression and/or upregulate glutamate production should be analyzed in future studies on *Pink1*−/− vocal behaviors and as vocal modulators.

In addition to GABA/glutamatergic systems, the gene enrichment analysis of the female vocalization module cyan (this module was significantly correlated to all USV parameters) suggest an important relationship between monoamine solute carriers and neuromodulators in female rat vocalization. From the enrichment analysis (visualized in Fig. 5), the vesicular monoamine transporter *Slc6a4* (transports serotonin) and *Ddc* (dopamine/serotonin) both directly interact with neurotensin (*Nts*). *Nts* is a neuromodulator that has been linked to social behavior and song production in birds, its mRNA expression is correlated to song production [62]. In rats, neurotensin agonists decreases stress induced 22-kHz vocalizations [63], but its effects on 50-kHz social vocalizations is unknown. Moreover, neurotensin shows significant relationships with other genes; for example, there are interactions with PAX transcription factor genes and neuron derived neurotrophic factor. These gene targets provide insight into the PAG-specific relationships with vocalization parameters and are promising therapeutic targets for observed decrease in vocal loudness in the *Pink1*−/− female rat model and may be translatable to the male acoustic dysfunction. In general, this work presents researchers with data mining opportunities, specifically to identify relationships between acoustic traits and any subset of these genes.

## Conclusions

The data suggest that loss of *Pink1* influences gene pathways within the brainstem PAG. Additionally, this study provides links and validation of this rat model as being useful for studying the disease as our data sets also match to human idiopathic Parkinson and PARK2 gene databases. These results indicate a key role for specific genes in vocal behavior and suggest potential drug targets for PD vocal deficits.

## Methods

### Rats, housing, and acclimation

A total of 16 rats were used in this study; 8 male (*n* = 4 *Pink1*−/−, n = 4 WT) and 8 female (n = 4 *Pink1*−/−, n = 4 WT) Long Evans rats (SAGE™ Research Labs, Boyertown, PA, USA) were aged to 8 months [25, 64]. Rats were housed in groups of two (within like genotypes/sex) in standard polycarbonate cages (290 mm × 533 mm × 210 mm) with corn cob bedding on a reversed 12:12 h light: dark cycle. All test procedures occurred during the dark period of the cycle. Food and water were available ad libitum*.* All procedures were approved by the University of Wisconsin-Madison Animal Care and Use Committee and were conducted in accordance with the National Institutes of Health Guide for the Care and Use of Laboratory animals [65].

### Female vocalization testing and analysis

A study of female-specific behavior was initially published in Marquis et al., 2019 [26]. Methods for data collection have been previously described [24, 58, 66]. Frequency-modulated acoustic (duration (s), bandwidth (Hz), intensity (loudness, dB), and peak frequency (Hz)) and non-acoustic variables (number of calls, percent of complex calls, call rate (calls/90 s)) were used in the statistical correlation analysis, discussed below.

### Tissue harvest and processing

Rats were deeply anesthetized with isoflurane and rapidly decapitated. The brains were dissected and immediately frozen and stored at − 80 °C. Brains were sliced coronally on a freezing cryostat at 250 μm thickness at − 15 °C and mounted on glass slides. Anatomically equivalent sections were used from each rat and 2 mm tissue punches (left and right) were collected within the PAG (Bregma ~ − 7.8 mm) using the Brain Punch Set (FST 18035–02, Foster City, CA, USA) as in previously reported methods [31]. Tissue samples were transferred to microcentrifuge tubes and stored at − 80 °C until processing for RNA.

### RNA preparation

Sample order was randomized throughout the molecular portion of the study. Brain tissue was homogenized with an electric sonic dismembrator (Fisher Scientific, Hampton, NH, USA) and was extracted using the Bio-Rad Aurum Total RNA Fatty and Fibrous Tissue Kit (Catalog No. 732–6830; Bio-Rad, Hercules, CA, USA). The total RNA was measured using a Nanodrop system (Thermo Scientific, Wilmington, DE, USA) and yielded significant concentrations of RNA. Additionally, the 28S:18S rRNA was quantified with an Agilent RNA 6000 Pico kit (Eukaryote Total RNA Pico, Agilent Technologies, Santa Clara, CA) and verified using a Agilent 2100 bioanalyzer (Agilent Technologies, Santa Clara, CA, USA). All samples had an A260/A280 ratio that fell within the 1.92–2.02 range and showed satisfactory marker and ribosomal peaks. RNA Integrity Numbers were above 7.2.

### Library construction and RNA sequencing

All RNA-sequencing procedures followed guidelines by ENCODE and were performed by the University of Wisconsin-Madison Biotechnology Center’s Next Generation Sequencing Facility. The Illumina® Total RNA-Seq TruSeq platform (Illumina Inc., San Diego, CA, USA) was used to profile differential expression of genes in the PAG between *Pink1*−/− and WT rats. Males (WT and *Pink1*−/−) and females (WT and *Pink1*−/−) were processed in separate batches, but preparation and processing of tissue as described above was identical. Briefly, the Stranded Total RNA Library Prep Kit was used to remove rRNA, and a sequencing library was generated. To prepare libraries, 500 ng was used as an input, rRNA reduction was done using H/M/R reagents (TruSeq Stranded Total RNA kit), samples were processed with RNA clean beads and 70% ethanol. The 3′ and 5′ adapters were ligated to small RNAs, followed by reverse transcription to obtain single-stranded complementary DNA. PCR-amplification used a universal primer and a primer containing a unique index sequence (Illumina, Inc). The amplified complementary DNA libraries were gel purified and used to construct RNA sequencing libraries. Libraries were quantified using Qubit DNA HS kit, diluted 1:100, assayed on Agilent DNA1000 chip. Libraries showed no adapter dimer contamination. Sequencing was performed on an HiSeq 2000 high-throughput sequencing system within a single run (Illumina, Inc). Adaptor sequences, contamination and low-quality reads were removed. Reads were mapped to the annotated rat (*Rattus norvegicus*) genome in Ensembl [67]. Technical quality was determined using several parameters. Briefly, trimming software skewer [68] was used to preprocess raw fastq files. Combined cycle base quality, per cycle base frequencies and average base quality, relative 3-Kmer diversity, Phred quality distribution, mean quality distribution, average read length and read occurrence distribution were used as additional quality control measures on the read data. Additionally, the biological coefficient of variation was estimated to be approximately 0.12. As such, a number of differentially expressed genes were identified, including *Pink1*−/− (Raw data (RSEM), is displayed in Supplementary Tables 6 and 7).

### Differential gene expression analysis and KEGG pathway enrichment

Analysis was performed with the glm using the EdgeR Bioconductor Package, v. 3.9 [69]. The *p*-value cutoff was set to 0.05 for significance. A Benjamini-Hochberg correction was applied to control the False Discovery Rate (FDR) [70]. Statistics and FDR for both male and female datasets (EdgeR Results) are provided in Supplementary Tables 8 and 9, respectively. RSEM approach for normalizing RNA seq data was used [71]; raw data were uploaded to the Gene Expression Omnibus (https://www.ncbi.nlm.nih.gov/geo/query/acc.cgi?acc=GSE150939; GSE150939). Genes were ranked according to *p* value and sorted by up- or down-regulation. The average LogCPM for all genes was similar for males (3.69) and females (3.92). The top 500 genes for males and females (both up- and down -regulated; see Supplementary Table 10) were compared to identify similar expression changes between the sexes in response to knockout (hypergeometric test). The cutoff of 500 was used as this is expected to capture significant biological changes in both directions and this cutoff is the same as used by GEO2Enrichr function to extract information from gene expression studies [72].

KEGG pathway enrichment was used to identify biological themes in the collection of differentially expressed genes. To conduct this test, all genes with a *p*-value < 0.05 were selected and for each pathway input genes that are part of the specific pathway were counted. The list of genes in every pathway was tested for over- or under-representation with respect to the input list of DE genes. The number of “background” genes was determined by counting the number of protein coding genes [Biotype] in the annotation model (described in [73].

### Weighted gene co-expression network (WGCNA) analysis

WGCNA was used to construct gene co-expression networks and gene modules from the gene expression datasets. Briefly, data were log 2 + 1 transformed, low expression genes were removed (specifically, using the filter function in EdgeR genes that had no expression in any of the individuals were removed) and WGCNA was run (on males: 13253 genes; females: 13277 genes; combined-sexes: 13253 genes) using R software (https://www.r-project.org/) [74]. Using a weighted network of genes and expression correlates (nodes and edges), correlations were raised to a soft thresholding power β of 12. Searchable networks were created for: [1] male rats, [2] female rats, and [3] combined-sexes (Supplementary Tables 1, 2, 3, 4 and 5).

Unsupervised hierarchical clustering for WGCNA included: the minimum module size of 30 genes, the signed mode, the deepSplit parameter set to 2, the mergeCutHeight parameter set to 0.15, and a threshold setting for merging modules of 0.25. Module eigengene values were also evaluated in terms of their genotype and vocal traits. Modules were visualized using Cytoscape (v3.7.1 48; https://cytoscape.org/) and the network file was exported and manually trimmed as to consist of genes of interest and the specific gene-to-gene correlations.

### Gene enrichment analysis

In order to identify genes that are over-represented in the data and associated with particular functions and relevance to PD, gene enrichment analysis was performed on both the differentially expressed gene dataset and the gene modules produced by the WGCNA analysis. Gene enrichment was performed with EnrichR [72].

### Protein-protein interactions

Genes dysregulated in *Pink1*−/− rats were compared with genes dysregulated in the same direction in human PD using multiple datasets (GSE7621, GSE19587, GSE17204) and the meta-analysis list in [33]. This human-rat list of 160 genes was entered into STRINGdp (v 2.0; Search Tool for the Retrieval of Interacting Genes/Proteins, http://string.embl.de/) to identify genes with high protein-protein interactions (Supplementary Table 11).

## Supplementary information

**Additional file 1: Table S1.** Gene modules identified by WGCNA in male rats.

**Additional file 2: Table S2.** Gene modules identified by WGCNA for female rats.

**Additional file 3: Table S3.** Gene modules identified by WGCNA for all rats.

**Additional file 4: Table S4.** Relationships between WGCNA identified modules and female vocal behaivor.

**Additional file 5: Table S5.** Relationships between WGCNA identified modules and female vocal behaivor.

**Additional file 6: Table S6.** Male RNA-Seq data used to analyze differential gene expression using EdgeR.

**Additional file 7: Table S7.** Female RNA-Seq data used to analyze differential gene expression using EdgeR.

**Additional file 8: Table S8.** Results of a differential gene expression in female rats.

**Additional file 9: Table S9.** Results of a differential gene expression in female rats.

**Additional file 10: Table S10.** Top 500 common up- and down-regulated genes in male and female Pink1−/− rats.

**Additional file 11: Table S11.** STRING Gene List.

## Data Availability

The datasets generated during the current study are available in the Gene Expression Omnibus repository (GSE150939). Website: https://www.ncbi.nlm.nih.gov/geo/query/acc.cgi?acc=GSE150939 The datasets analyzed during the current study are available in the Gene Expression Omnibus repositories (GSE7621, GSE19587, GSE17204) and as described in Table 1 as well as the annotated rat genome (Rat Rnor_6.0 assembly; https://www.ebi.ac.uk/ena/browser/view/GCA_000001895.4).

## Abbreviations

PD: Parkinson’s disease
PAG: periaqueductal gray
WT: wildtype
s: seconds
Hz: Hertz
kHz: Kilo-hertz
dB: Decibels
